# Patterns and Outcomes of Surgically Treated Non-traumatic Acute Abdomen in a Teaching Hospital in Nigeria: A 10-Year Retrospective Study

**DOI:** 10.7759/cureus.110608

**Published:** 2026-06-10

**Authors:** Oluwatosin G Afolabi, Adedamola B Adegbamigbe, Mayowa E Oluwajuyigbe, Abiodun I Okunlola

**Affiliations:** 1 General Surgery, Federal Teaching Hospital, Ido Ekiti, NGA; 2 Neurosurgery Unit, Department of Surgery, Afe Babalola University, Ado Ekiti, NGA

**Keywords:** low-income setting, nigeria, non-traumatic acute abdomen, outcomes, patterns

## Abstract

Aim/objective: This study aimed to investigate the epidemiology, clinical patterns, and outcomes of surgically treated non-traumatic acute abdomen in a Nigerian teaching hospital, identifying key predictors of morbidity and mortality.

Materials and methods: This retrospective study, conducted from 2014 to 2024 at Federal Teaching Hospital, Ido-Ekiti, Nigeria, included 611 patients undergoing surgery for non-traumatic acute abdomen. Obstetric, gynecological, pediatric, traumatic, or conservatively managed cases were excluded. Data on demographics, diagnoses, complications, and outcomes were collected from medical records and analyzed using IBM SPSS Statistics for Windows, version 21.0. Logistic regression identified predictors of adverse outcomes, with significance set at p < 0.05.

Results: Non-traumatic acute abdomen accounted for 16.94% (611/3,605) of surgical operations. The cohort (51.06% male, 312/611; 48.93% female, 299/611; mean age 39.70 years) predominantly comprised younger patients (18-27 years, 35.19%, 215/611). Acute appendicitis (51.06%, 312/611), intestinal obstruction (30.61%, 187/611), and perforation (13.75%, 84/611) were the leading diagnoses. Complications occurred in 21.77% (133/611) of cases, primarily wound infection (29.29%, 39/133), sepsis (13.74%, 18/133), and chest infection (9.00%, 12/133). Mortality was 9.98% (61/611), mainly due to septic shock (52.46%, 32/61) and fecal fistula (19.67%, 12/61). Fever, comorbidities, delayed presentation (>7 days), and referred status were significant predictors of adverse outcomes, as detailed in the multivariate analysis.

Conclusion: Non-traumatic acute abdomen carries a high burden in Nigeria, driven largely by appendicitis, bowel obstruction, and visceral perforation. Outcomes are worsened by delays at several points along the care pathway, from late recognition of symptoms and delayed decisions to seek care, to prolonged transit to facilities and protracted waits for diagnosis and surgery once patients arrive. These cumulative delays, compounded by systemic infection and coexisting comorbidities, escalate morbidity and mortality, underscoring the need for earlier presentation, faster diagnostic access, and timely surgical intervention.

## Introduction

Acute abdomen is a common surgical emergency associated with high morbidity and mortality if not managed properly [1]. It represents the most frequent presenting surgical emergency, with an estimated 50% of general surgical admissions being emergencies, of which half present with acute abdominal pain [2]. Acute abdomen refers to sudden-onset, severe abdominal pain requiring urgent medical or surgical evaluation [1]. This clinical syndrome encompasses a wide range of conditions, including appendicitis, intestinal obstruction, perforated peptic ulcers, and acute pancreatitis, with the distribution of diagnoses varying by age and sex [1]. The attending emergency physician faces significant challenges in identifying the underlying cause, particularly in resource-limited settings where diagnostic and management capacities may be constrained.

In African countries, the pattern of acute abdomen is evolving, likely owing to shifts toward low-residue diets, modern lifestyles, and increasing societal stress [3,4]. Historically, before the widespread use of modern imaging techniques such as ultrasound and computed tomography (CT), most patients diagnosed with an acute abdomen underwent surgical exploration [5,6]. However, current imaging modalities have improved diagnostic accuracy, allowing many patients with non-specific abdominal pain to be managed conservatively [6,7]. Despite these advances, intestinal obstruction remains the leading cause of acute abdomen in several African countries, primarily due to hernia and volvulus, whereas adhesions predominate in developed countries [8-11]. Conversely, acute appendicitis is the most common cause in the developed world [10,11]. Recent African studies suggest a shift in these established patterns, underscoring the need for updated epidemiological data [12,13].

In Nigeria, teaching hospitals serve as critical referral centers for managing complex surgical cases, including non-traumatic acute abdomen. However, there is a paucity of comprehensive, up-to-date data on the patterns, management strategies, and outcomes of these conditions in such settings [14]. The most recent published study on acute abdomen in Nigeria was conducted over a decade ago and is now outdated [7]. This scarcity of current data limits efforts to optimize resource allocation, enhance surgical training, and improve patient outcomes in resource-constrained environments [15]. Understanding the epidemiology, clinical presentations, and surgical outcomes is essential for addressing these challenges and informing health policy.

This 10-year retrospective study aims to assess the magnitude, patterns, and outcomes of surgically treated non-traumatic acute abdomen at Federal Teaching Hospital, Ido-Ekiti, Nigeria. By analyzing patient demographics, clinical diagnoses, surgical interventions, and postoperative outcomes over a decade, the study seeks to identify prevalent conditions, evaluate treatment efficacy, and highlight factors influencing morbidity and mortality. The findings will be compared with those of other studies in Nigeria, sub-Saharan Africa, and the developed world. This study is expected to provide epidemiological and clinical insights, serve as a foundation for future research, and contribute to improving surgical care delivery in Nigeria’s resource-limited settings.

## Materials and methods

This was a 10-year retrospective study conducted at Federal Teaching Hospital, Ido-Ekiti, Nigeria, spanning from January 2014 to December 2024. The hospital is a multi-specialty referral center and one of three tertiary hospitals in Ekiti State, located in southwestern Nigeria. It serves as a key facility for managing complex surgical cases in the region.

Eligibility criteria

Inclusion Criteria

The study population included all patients who underwent surgical operations for a preliminary diagnosis of non-traumatic acute abdomen during the study period. Patients were eligible if they had a confirmed surgical intervention for conditions such as appendicitis, intestinal obstruction, perforated peptic ulcers, or other non-traumatic acute abdominal pathologies requiring operative management.

Exclusion Criteria

Patients were excluded from the study if they underwent obstetric or gynecological surgeries, required reoperation due to postoperative complications such as stoma prolapse, enterocutaneous fistula, or burst abdomen closure, were pediatric patients (typically defined as under 18 years of age, unless otherwise specified by the hospital’s protocol), presented with traumatic acute abdomen, had non-specific acute abdominal pain managed conservatively without surgical intervention, or had incomplete medical records or records that could not be retrieved from the hospital’s records department.

Data collection and analysis

Data were retrieved from theatre records and patient case notes at Federal Teaching Hospital, Ido-Ekiti, Nigeria’s records department, for 10 years (January 2014-December 2024). Extracted variables included patient demographics (age and sex), clinical findings (e.g., fever and duration of presentation), intraoperative diagnoses, postoperative complications, and clinical outcomes (morbidity and mortality). Patients with obstetric, gynaecological, pediatric, traumatic, or conservatively managed cases or incomplete records were excluded, resulting in 611 eligible cases. Data were entered and analysed using IBM SPSS Statistics for Windows, version 21.0 (released 2012, IBM Corp., Armonk, NY). Descriptive statistics included frequencies, means, proportions, and summarized demographic and clinical characteristics. Multivariate logistic regression was used to identify predictors of postoperative morbidity and mortality, calculating odds ratios (ORs), 95% confidence intervals (CIs), and chi-square values. Statistical significance was defined as p < 0.05. Results were presented in tables to illustrate patterns, operative findings, complications, and outcomes of surgically treated non-traumatic acute abdomen.

## Results

**Figure 1:**
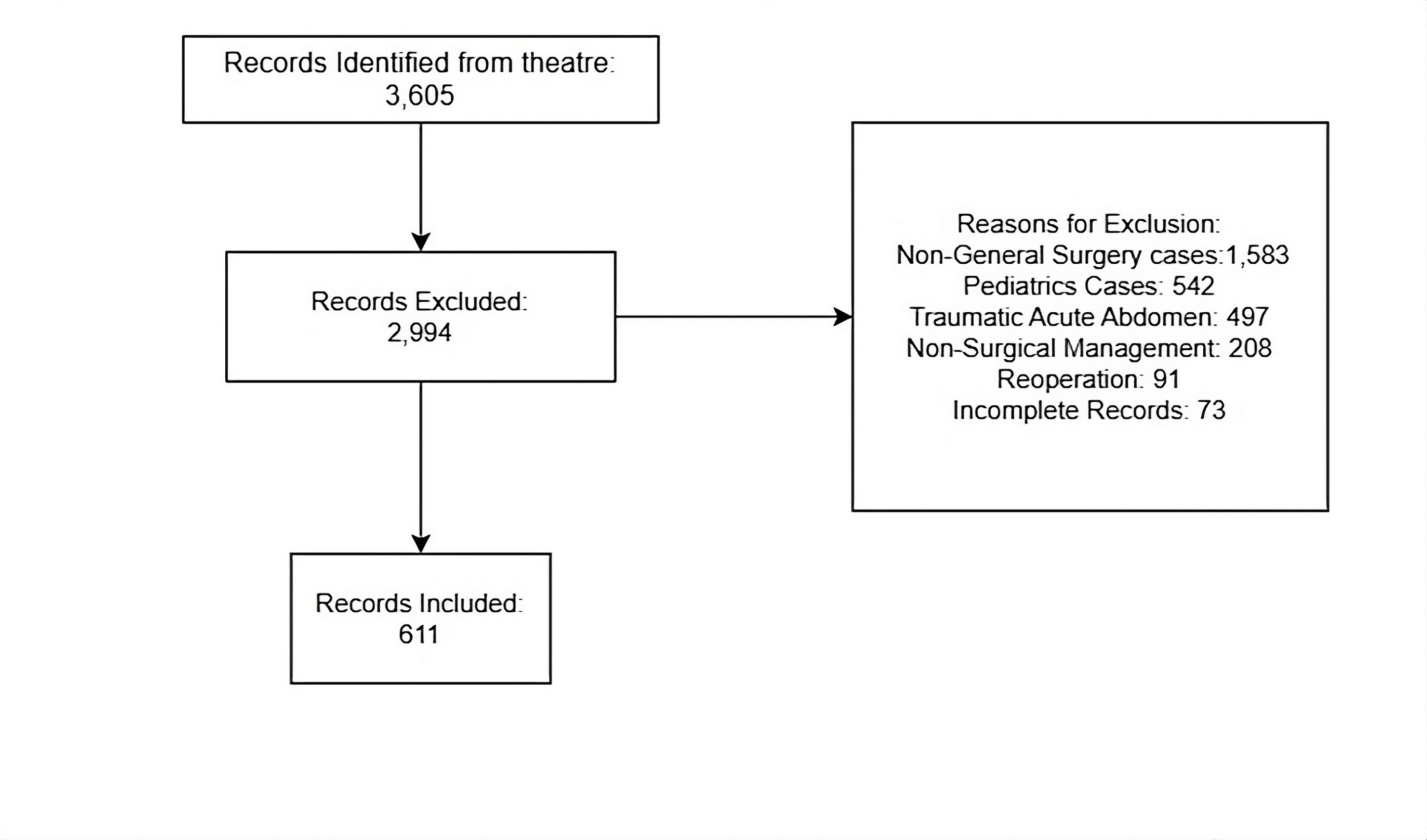
Flow chart of record screening based on the eligibility criteria

During the study period from January 2014 to December 2024, a total of 3,605 surgical operations were performed at Federal Teaching Hospital, Ido-Ekiti, Nigeria. Of these, 2,994 operations (83.06%) were excluded based on the criteria outlined in the flowchart, primarily owing to obstetric or gynecological procedures, reoperations for complications, pediatric cases, traumatic acute abdomen, non-specific abdominal pain managed conservatively, or incomplete or irretrievable records. Consequently, 611 operations (16.94%) met the inclusion criteria and constituted the study population (Figure 1).

Patient demographics

**Figure 2:**
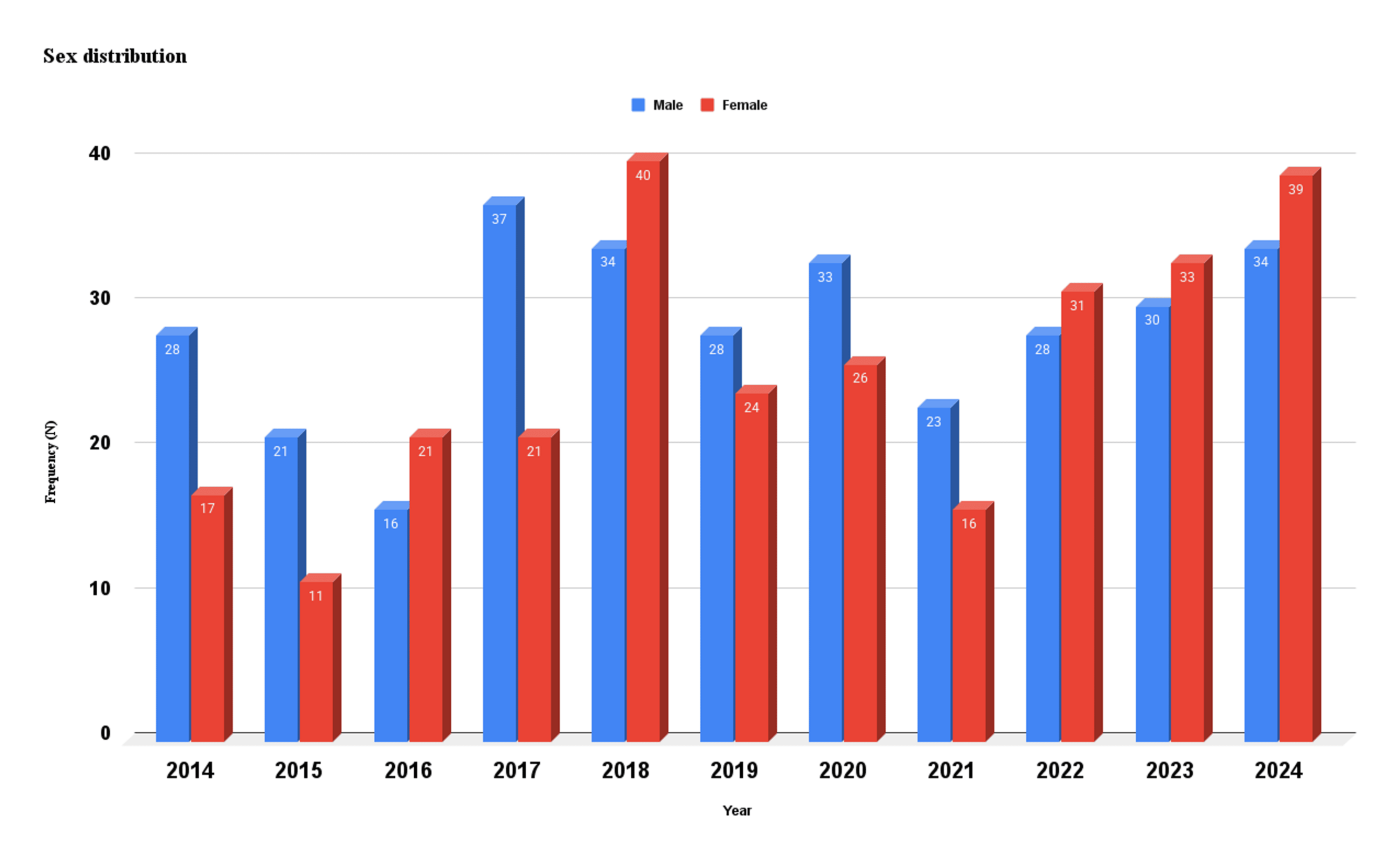
Sex distribution of the study population

**Figure 3:**
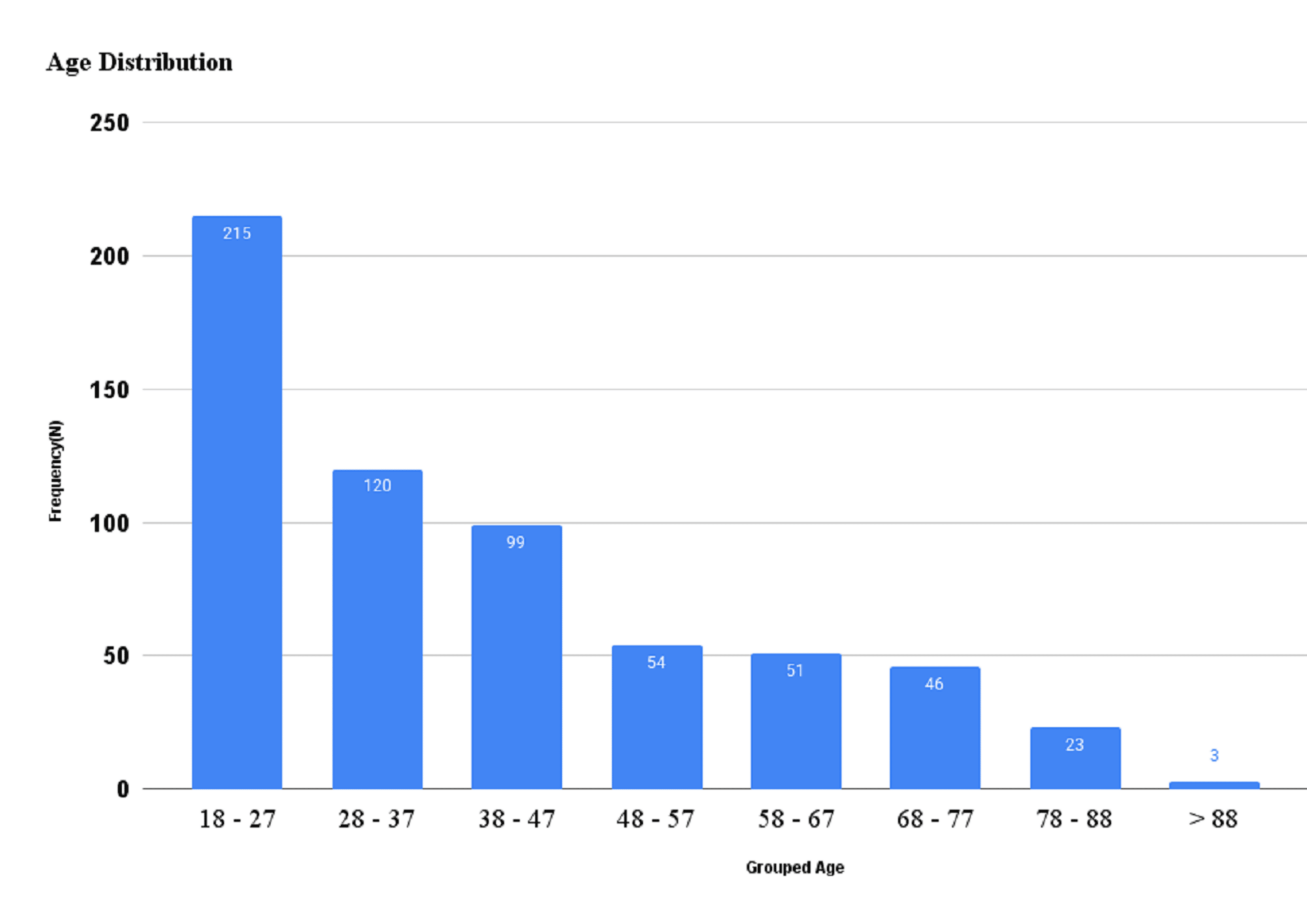
Age distribution of the study population

The study population comprised 611 patients, with 312 (51.06%) males and 299 (48.93%) females, yielding a male-to-female ratio of 1.04:1 (Figure 2). Patient ages ranged from 18 to 92 years, with a mean age of 39.70 years (SD ± 4.71). The age distribution showed two peak groups: 18-27 years (215 patients, 35.19%) and 28-37 years (120 patients, 19.64%), as illustrated in Figure 2. The remaining age groups were distributed as follows: 38-47 years (99 patients, 16.20%), 48-57 years (54 patients, 8.84%), 58-67 years (51 patients, 8.35%), 68-77 years (46 patients, 7.53%), 78-88 years (23 patients, 3.76%), and >88 years (three patients, 0.49%) (Figure 3).

Clinical features

The clinical features are summarized in Table 1. All 611 patients (100%) presented with abdominal pain. Other common symptoms included vomiting (473, 77.40%), constipation (291, 47.62%), and abdominal distension (209, 34.20%). The duration of symptoms before presentation was less than three days in 294 patients (48.11%), three to seven days in 203 (33.22%), and more than seven days in 114 (18.65%). On examination, abdominal tenderness was noted in 523 patients (85.59%), generalized tenderness in 366 (59.90%), rebound tenderness in 244 (39.93%), and shock in 101 (16.53%).

**Table 1:** Clinical symptoms and signs Data are presented as frequency (N) and percentage (%).

Clinical features	Frequency N, (%)
Duration of symptoms before presentation	
<3 days	294 (48.11)
3-7 days	203 (33.22)
>7 days	114 (18.65)
Symptoms	
Abdominal pain	611 (100)
Vomiting	473 (77.4)
Constipation	291 (47.62)
Abdominal distention	209 (34.20)
Signs	
Localized tenderness	523 (85.59)
Rebound tenderness	244 (39.93)
Generalized tenderness	366 (59.90)
Fever	283 (46.32)
Shock	101 (16.53)

Operative diagnosis

The operative diagnoses and their frequencies are presented in Table 2. Acute appendicitis was the most common diagnosis, accounting for 312 cases (51.06%), of which 234 (38.29%) were classified as simple appendicitis treated with open appendectomy and 78 (12.76%) as complex appendicitis (appendiceal mass: 23, appendiceal abscess: 22, ruptured appendix: 33) requiring exploratory laparotomy. Slightly more males than females were diagnosed with acute appendicitis (male-to-female ratio 1.9:1). Simple appendicitis occurred predominantly in patients in their first and second decades, whereas 73 (93.58%) of complex appendicitis cases occurred in patients over 40 years, with only five (6.42%) under 40 years.

**Table 2:** Operative diagnosis Data are presented as frequency (N) and percentage (%).

Operative diagnosis	Frequency, N (%)
Appendicitis	
Simple (open appendectomy)	234 (38.29)
Complex (exploratory laparotomy)	78 (12.76)
Intestinal obstruction	
Hernia	49 (8.02)
Adhesions	25 (4.09)
Small bowel volvulus	14 (2.29)
Gastric outlet obstruction	26 (4.25)
Colorectal cancer	44 (7.20)
Pancreatic cancer	11 (1.80)
Sigmoid volvulus	18 (2.95)
Intestinal perforation	
Typhoid perforation	28 (4.58)
Peptic ulcer disease	36 (5.89)
Small bowel (others)	12 (1.96)
Large bowel	8 (1.31)
Gallbladder disease	11 (1.80)
Intra-abdominal abscess	
Splenic abscess	15 (2.45)
Subphrenic abscess	2 (0.33)
Total	611

Intestinal obstruction accounted for 187 cases (30.61%), including hernia (49, 8.02%), adhesions (25, 4.09%), small bowel volvulus (14, 2.29%), gastric outlet obstruction (26, 4.25%), colorectal cancer (44, 7.20%), pancreatic cancer (11, 1.80%), and sigmoid volvulus (18, 2.95%). Among hernias, 37 (75.51%) were umbilical or paraumbilical and presented as strangulated or obstructed. Ninety-six percent of gastric outlet obstruction cases occurred in senior citizens (age ≥60 years) and were due to gastric cancer. Large bowel obstruction was caused primarily by colorectal cancer (left-sided: 28, 63.64%; right-sided: nine, 20.45%; rectal: seven, 15.91%), with only six patients under 40 years, and by sigmoid volvulus.

Intestinal perforation totaled 84 cases (13.75%), with peptic ulcer disease (PUD) (36, 5.89%) and typhoid ileal perforation (28, 4.58%) as the leading causes. Other small bowel perforations (12, 1.96%) were attributed to tuberculosis-related perforation and ischemic bowel disease, both common in low-income settings. Large bowel perforations (eight, 1.31%) were caused by amoebic colitis and diverticular disease. Typhoid ileal perforation predominantly affected young adults (age 18-33 years), whereas 91.67% of PUD perforation cases occurred in chronic nonsteroidal anti-inflammatory drug (NSAID) users.

Gallbladder disease was diagnosed in 11 cases (1.80%), all in females aged 40 years or older, with cholecystitis (nine, 81.82%) and choledocholithiasis (two, 18.18%) as the causes. Intra-abdominal abscesses included splenic abscess (15, 2.45%) and subphrenic abscess (two, 0.33%). Of the splenic abscess cases, 12 (80%) occurred in patients with sickle cell disease, whereas the other intra-abdominal abscesses were associated with diabetes and other systemic illnesses.

Complications and mortality

Complications are presented in Table 3. The most common morbidity was wound infection (179, 29.29%), followed by sepsis (84, 13.74%), chest infection (55, 9.00%), burst abdomen (31, 5.07%), anastomotic leak (29, 4.75%), and fecal fistula (19, 3.11%). The overall mortality rate was 9.98% (61 deaths). Causes of mortality included septic shock (32, 52.46%), cardiopulmonary complications (nine, 14.75%), fecal fistula (12, 19.67%), and cachexia (eight, 13.11%). Of the 312 patients with appendiceal disease, 11 (3.53%) died from overwhelming sepsis, all of whom presented late. Deaths from fecal fistula occurred primarily among patients with typhoid ileal perforation (five cases) and gangrenous bowel due to intestinal obstruction. All patients who died from cachexia with severe malnutrition had malignancies (colorectal or pancreatic cancer).

**Table 3:** Postoperative complications (morbidity and mortality) Data are presented as frequency (N) and percentage (%).

Complications	Frequency, N (%)
Wound infection	179 (29.9)
Chest infection	55 (9.0)
Burst abdomen	31 (5.07)
Sepsis	84 (13.7)
Fecal fistula	19 (3.11)
Anastomotic leak	29 (4.75)
Mortality	
Septic shock	32 (52.45)
Cardiopulmonary	9 (14.75)
Fistula	12 (19.67)
Cachexia	8 (13.11)
Overall mortality	61 (100)

Predictors of outcome

Morbidity

Multivariate analyses of the predictors of outcome are shown in Tables 4-5. Fever at presentation was the strongest predictor of morbidity, with 93.85% of patients with fever (122/130) developing complications. This high odds ratio (OR 36.07) suggests that fever is a critical indicator of advanced disease, such as peritonitis or systemic infection, likely exacerbated by limited diagnostic resources or delayed access to care in Nigeria. The presence of comorbidity was another significant risk factor, with 61.58% of patients with comorbidities (109/177), such as sickle cell disease or diabetes, developing complications. This underscores the challenge of managing underlying health conditions in a resource-constrained setting, where preoperative optimization may be inadequate.

**Table 4:** Multivariate analysis of predictors of morbidity rates Data are presented as frequency (N) and percentage (%). Logistic regression was used to calculate odds ratios (ORs) and 95% confidence intervals (CIs). P-values were derived from chi-square tests within logistic regression models. Statistical significance is defined as p < 0.05.

Predictors	Patients, N (%)	Morbidity, N (%)	Morbidity rate (%)	OR	95% CI	Chi-square	P-value
Age							
18-27	215 (35.19%)	29 (21.80%)	13.49%	0.37	0.24-0.57	20.32	<0.001
28-37	120 (19.64%)	22 (16.54%)	18.33%	0.59	0.36-0.98	4.12	0.042
38-47	99 (16.19%)	17 (12.78%)	17.17%	0.54	0.31-0.93	4.89	0.027
48-57	54 (8.84%)	16 (12.03%)	29.63%	1.22	0.66-2.55	0.40	0.529
58-67	51 (8.35%)	19 (14.29%)	37.25%	1.83	1.00-3.35	3.84	0.049
68-77	46 (7.53%)	18 (13.53%)	39.13%	1.99	1.06-3.74	4.55	0.033
89-88	23 (3.76%)	10 (7.52%)	43.48%	2.39	1.02-5.60	3.97	0.046
>88	3 (0.49%)	2 (1.50%)	66.67%	5.91	0.53-65.58	2.06	0.151
Presence of comorbidity							
Yes	177 (28.97%)	109 (81.95%)	61.58%	12.15	7.64-19.33	96.47	<0.001
No	434 (71.03%)	24 (18.05%)	5.53%	0.08	0.05-0.13	96.47	<0.001
Duration of presentation							
<3 days	294 (48.12%)	29 (21.80%)	9.86%	0.24	0.16-0.37	37.12	<0.001
3-7 days	203 (33.22%)	48 (36.09%)	22.65%	0.84	0.57-1.25	0.73	0.391
>7 days	114 (18.66%)	62 (46.62%)	54.39%	5.11	3.35-7.80	53.79	<0.001
Presentation							
Direct	445 (72.83%)	42 (31.58%)	9.44%	0.23	0.15-0.34	46.53	<0.001
Referred	166 (27.17%)	63 (47.37%)	37.95%	3.45	2.28-5.21	34.27	<0.001
Fever at presentation							
Yes	130 (21.28%)	122 (91.73%)	93.85%	36.07	18.66-69.76	97.63	<0.001
No	481 (78.72%)	11 (8.27%)	2.29%	0.03	0.01-0.05	97.63	<0.001
Hospital stay duration							
<5 days	375 (61.37%)	36 (27.07%)	9.60%	0.24	0.16-036	37.05	<0.001
5-10 days	139 (22.75%)	55 (41.35%)	39.57%	2.31	1.54-3.47	13.82	<0.001
>10 days	97 (15.88%)	42 (31.58%)	43.30%	2.64	1.71.409	18.05	<0.001

**Table 5:** Multivariate analysis of the predictors of morbidity rates Data are presented as frequency (N) and percentage (%). Logistic regression was used to calculate odds ratios (ORs) and 95% confidence intervals (CIs). P-values were derived from chi-square tests within logistic regression models. Statistical significance is defined as p < 0.05.

Predictors	Patients, N (%)	Mortality, N (%)	Mortality rate (%)	OR	95% CI	Chi-square	P-value
Age							
18-27	215 (35.19%)	5 (8.20%)	2.33%	0.15	0.06-0.39	14.76	<0.001
28-37	120 (19.64%)	7 (11.48%)	5.83%	0.46	0.20-1.04	3.46	0.063
38-47	99 (16.19%)	12 (19.67%)	12.12%	1.37	0.74-2.67	0.85	0.357
48-57	54 (8.84%)	10 (16.39%)	18.52%	2.39	1.14-5.02	5.30	0.021
58-67	51 (8.35%)	11 (18.03%)	21.57%	2.97	1.43-6.17	8.60	0.003
68-77	46 (7.53%)	8 (13.11%)	17.39%	2.22	0.98-5.02	3.68	0.055
789-88	23 (3.76%)	6 (9.84%)	26.09%	3.79	1.46-9.86	7.46	0.006
>88	3 (0.49%)	2 (3.28%)	66.67%	19.62	1.76-218.81	5.76	0.016
Presence of comorbidity							
Yes	177 (28.97%)	42 (66.85%)	22.73%	7.62	4.32-13.53	45.37	<0.001
No	434 (71.03%)	19 (31.15%)	4.38%	0.13	0.07-0.23	45.37	<0.001
Duration of presentation							
<3 days	294 (48.12%)	11 (18.03%)	3.75%	0.22	0.11-0.43	16.92	<0.001
3-7 days	203 (33.22%)	17 (27.87%)	8.37%	0.75	0.41-1.36	0.90	0.341
>7 days	114 (18.66%)	33 (54.10%)	28.95%	6.61	3.79-11.52	39.31	<0.001
Presentation							
Direct	445 (72.83%)	28 (45.90%)	6.29%	0.34	0.20=0.58	14.93	<0.001
Referred	166 (27.17%)	33 (54.10%)	19.88%	3.67	2.15-6.27	20.55	<0.001
Fecal fistula							
Yes	19 (3.11%)	12 (19.67%)	63.16%	18.98	7.76-46.01	34.88	<0.001
No	592 (96.89%)	49 (80.33%)	8.28%	0.05	0.02-0.13	34.88	<0.001
Sepsis							
Yes	84 (13.75%)	32 (52.46%)	38.10%	10.58	5.94-18.9	55.13	<0.001
No	527 (96.89%)	29 (47.54%)	5.50%	0.09	0.05-0.17	55.13	<0.001
Fever at presentation							
Yes	130 (21.28%)	42 (68.85%)	32.31%	8.27	4.69-14.59	45.47	<0.001
No	481 (78.72%)	19 (31.15%)	3.95%	0.12	0.07-0.21	45.47	<0.001
Hospital stay duration							
<5 days	375 (61.37%)	8 (13.11%)	2.13%	0.13	0.06-0.27	24.55	<0.001
5-10 days	139 (22.75%)	17 (27.87%)	12.33%	1.36	0.75-2.46	1.05	<0.305
>10 days	97 (15.88%)	36 (59.02%)	37.11%	6.92	3.92-12.23	37.85	<0.001

Delayed presentation (>7 days) markedly increased morbidity risk, with a morbidity rate of 54.39%, whereas presentation within three days was protective (9.86% morbidity rate). This highlights the critical role of timely surgical intervention in preventing complications such as wound infection or sepsis, which are prevalent in prolonged disease states. Referred patients had a higher morbidity risk (37.95% morbidity rate) than direct presentations (9.44% morbidity rate), suggesting that delays in referral pathways or the severity of referred cases contribute significantly to adverse outcomes.

Age showed a graded effect on morbidity. Younger patients aged 18-27 years were less likely to develop complications (13.49% morbidity rate), whereas older patients aged 58-67 years had an increased risk, with the >88 years' group showing a 66.67% morbidity rate. However, the wide confidence interval for the >88 years' group (0.53-65.58, p = 0.151) and the small sample size (n = 3) limit the precision of this estimate, indicating that results for the oldest age group should be interpreted with caution. This age-related trend may reflect reduced physiological reserve or more complex underlying conditions in older patients, such as colorectal cancer or complicated appendicitis, which were prevalent in those over 40 years.

Prolonged hospital stays (>5 days) were associated with higher morbidity, whereas stays <5 days were protective. The 43.30% morbidity rate for stays >10 days likely reflects more severe conditions or postoperative complications requiring extended care, such as wound infections or anastomotic leaks, which prolong recovery.

Mortality

The overall mortality rate was 9.98% (61/611), primarily due to septic shock (52.46%) and fecal fistula (19.67%). Fecal fistula was the strongest predictor of mortality, with 63.16% of affected patients dying, reflecting the severe prognosis of this complication, which was often linked to typhoid perforation or gangrenous bowel in our cohort. Sepsis was another major risk factor, with a mortality rate of 38.10%, highlighting the lethal impact of systemic infection in a setting with limited intensive care resources.

Fever at presentation significantly increased mortality risk (32.31% mortality rate), likely indicating advanced disease at admission, such as peritonitis from perforated PUD or typhoid. The presence of comorbidity also elevated mortality risk (23.73% mortality rate), underscoring the burden of conditions, such as sickle cell disease, which was associated with splenic abscesses in our study.

Delayed presentation (>7 days) was a strong predictor of mortality (28.95% mortality rate), whereas presentation within 3 days was protective (3.74% mortality rate). Similarly, hospital stays >10 days increased mortality risk (OR 6.92, 37.11% mortality rate), whereas stays <5 days were protective (OR 0.13, 13.11% mortality rate). These findings suggest that early intervention and shorter hospital stays are associated with better outcomes, likely owing to less severe disease or fewer complications.

Referred patients had a higher mortality risk than direct presentations, possibly reflecting delays in reaching tertiary care or the complexity of referred cases, such as those with colorectal cancer or perforations. Age was a significant predictor in older groups (58-67 years: OR 2.97; 78-88 years: OR 3.79), with the >88 years group showing a 66.67% mortality rate, although the small sample size (n = 3) widens the confidence interval (1.76-218.81), indicating uncertainty.

## Discussion

The incidence of non-traumatic acute abdomen at Federal Teaching Hospital, Ido-Ekiti, Nigeria, was 16.94% (611/3,605 surgical operations), higher than the 9.6% reported at the University of Ilorin Teaching Hospital (UITH), Nigeria [7], but closely aligned with the 16.7% and 11.4% reported in Nairobi, Kenya, and Ethiopia, respectively [16,17]. The exclusion of gynecological and medical emergencies in our study, unlike Awori et al. [16], who included such cases, may account for the higher incidence compared with UITH, as their inclusion could inflate reported rates. However, our incidence is notably lower than the 36.4% reported in Ethiopia by Kotiso et al. [18], suggesting regional variations in surgical emergency burdens, potentially owing to differences in healthcare access, diagnostic delays, or disease prevalence. The near-equal sex distribution (51.06% male, 48.93% female; male-to-female ratio 1.04:1) contrasts with the higher male predominance in Ethiopia (2:1 [18]; 77.97% male [2]). This closer sex ratio in our study may reflect improved access to surgical care for females in our setting, possibly owing to enhanced healthcare infrastructure or awareness compared with other regional studies. The age distribution, peaking at 18-27 years (35.19%) and 28-37 years (19.64%), aligns with findings from multiple studies [2,12,18,19,20], in which patients in their second and third decades were most affected, reflecting the younger demographic affected by acute abdominal conditions in low-income settings.

Acute appendicitis was the leading cause of non-traumatic acute abdomen, accounting for 51.06% of cases (312/611), with a male-to-female ratio of 1.9:1. This finding is consistent with studies in Nigeria [7,12] and Ethiopia [2,18], in which appendicitis was the most common diagnosis (30.4% at UITH [7]; the leading cause in Ayenew’s and Kotiso’s series [2,18]). The male-to-female ratio for appendiceal disease in our study (1.9:1) is higher than Ajao’s report in Ibadan [13] but lower than UITH’s 3.4:1 [7], indicating variability in sex distribution, possibly influenced by regional differences in presentation or diagnostic practices. Simple appendicitis predominated in younger patients (first and second decades), consistent with Agboola et al.’s finding that 50% of appendicitis cases occurred in patients under 25 years [7]. By contrast, complex appendicitis (e.g., appendiceal mass, abscess, or rupture) was more prevalent in patients over 40 years (93.58%), a pattern not explicitly highlighted in other studies but consistent with delayed presentation in older age groups in low-income settings.

Intestinal obstruction was the second most common diagnosis (30.61%, 187/611), driven primarily by umbilical or paraumbilical hernias (75.51% of hernia cases) and colorectal cancer. This contrasts with UITH, where anterior abdominal wall hernias and postoperative adhesions (31.2%) were the leading causes of obstruction [7]. In Sokoto, Mbah et al. [21] reported intestinal obstruction as the primary cause of acute abdomen, unlike our appendicitis-dominated findings, potentially owing to dietary or socioeconomic factors [20]. Adhesions were less significant in our study (4.09%) than at UITH [7] or Gondar University Hospital, where adhesions were the primary cause of small bowel obstruction [22]. However, our findings align with Kotiso et al.’s report of small bowel adhesions (27.4%) as a significant cause in Ethiopia [18], although sigmoid volvulus was equally prevalent in their series. The prominence of sigmoid volvulus in Ayenew et al.’s study as the leading cause of colonic obstruction [2] is consistent with our sigmoid volvulus cases (2.95%). Intestinal perforation (13.75%, 84/611) was attributed primarily to PUD (5.89%) and typhoid ileal perforation (4.58%), with tuberculosis, ischemic bowel disease (1.96%), amoebic colitis, and diverticular disease (1.31%) as other causes. These etiologies reflect common patterns in low-income settings [7,12]. Compared with other studies, PUD perforation rates varied (9% [18]; 2.2% [20]), whereas typhoid perforation was twice as high in Ayenew et al.’s study [2]. The predominance of typhoid perforation in young adults (18-33 years) and the association of PUD perforation with chronic NSAID use (91.67%) highlight socioeconomic challenges, such as over-the-counter NSAID availability and limited healthcare access. Gallbladder disease (1.80%), exclusively cholecystitis in females over 40 years (81.82%), was less frequent than appendicitis or obstruction, consistent with its lower prevalence in African studies [7,12,18,20-26]. Splenic abscesses (2.45%), predominantly in patients with sickle cell disease (80%), reflect regional disease burdens, particularly in Nigeria, where sickle cell disease is prevalent.

The postoperative complication rate was 21.77% (133/611), comparable to that of Kotiso et al. (25.97%) [18] and Nega (22.9%) [27], but higher than Ademe et al.’s 14.8% [24]. Wound infection (29.29%), sepsis (13.74%), and chest infection (9.00%) were the most common complications, exceeding the rates reported by Ayenew et al. (7.8%, 2.3%, and 3.0%, respectively) [2] but aligning with those at Gondar University Hospital (20.6%, 17.6%, and 9.9%) [22]. The higher complication rates in our study compared with Ayenew et al. [2], but lower than Kotiso et al. [18] (28%), may reflect differences in surgical techniques, postoperative care, or patient comorbidities. Strong predictors included fever at presentation (OR 36.07, 95% CI: 18.66-69.76, p < 0.001) and comorbidities (OR 12.15, 95% CI: 7.64-19.33, p < 0.001), such as sickle cell disease in 80% of splenic abscess cases. Delayed presentation (>7 days) significantly increased morbidity (OR 5.11, 95% CI: 3.35-7.80, p < 0.001; 54.39% morbidity rate), consistent with Ademe et al.’s findings [24].

The overall mortality rate was 9.98% (61/611), similar to Nega’s 8.8% [27] and Gondar’s 9.3% [22], but lower than Kotiso et al.‘s 15.58% [18], Yirgalem’s 13.5% [20], Port Harcourt’s 13.3% [19], and Tikur Anbessa’s 14.0% [18], and higher than Ayenew et al.’s 3.0% [2] and Ademe et al.’s 1.9% [24]. Appendicitis had a lower mortality rate (3.53%), consistent with Kotiso et al. [18], whereas intestinal obstruction (e.g., gangrenous bowel) and perforation (e.g., typhoid) had higher rates, although less severe than Kotiso et al.’s 21.2% for small bowel obstruction and 35.2% for sigmoid volvulus [18]. Key predictors of mortality included fecal fistula (OR 18.98, 95% CI: 7.76-46.01, p < 0.001; 63.16% mortality rate), sepsis (OR 10.58, 95% CI: 5.94-18.89, p < 0.001; 38.10% mortality rate), and fever at presentation (OR 8.27, 95% CI: 4.69-14.59, p < 0.001). Delayed presentation (>7 days) increased mortality risk (OR 6.61, 95% CI: 3.79-11.52, p < 0.001; 28.95% mortality rate), whereas early presentation (<3 days) was protective (OR 0.22), aligning with Ademe et al.’s findings [24].

The mean postoperative hospital stay was 6.30 ± 4.71 days, longer than Ademe et al.‘s 3.3 ± 2 days [24] but shorter than Nega’s 8.74 days and McConkey’s 13.5 days [25,26], which included preoperative periods. Prolonged stays (>10 days) were associated with increased morbidity (OR 2.64) and mortality (OR 6.92, p < 0.001), particularly in complex appendicitis and colorectal cancer cases, exacerbated by complications such as wound infection (29.29%) and sepsis (13.74%). The association between delayed presentation and longer hospital stays aligns with Ademe et al.’s finding of a positive correlation (OR 1.59, 95% CI: 1.03-2.45) [24], highlighting the impact of delayed care on recovery time.

Delayed presentation (>7 days) significantly increased morbidity (OR 5.11, 95% CI: 3.35-7.80, p < 0.001) and mortality (OR 6.61, 95% CI: 3.79-11.52, p < 0.001), whereas early presentation (<3 days) was protective (OR 0.24 for morbidity, OR 0.22 for mortality). Kotiso et al. noted lower mortality for patients presenting within 2 days (7.6%) compared with those presenting after 2 days (25%) [18], underscoring the role of timely care. Referred patients had higher morbidity (OR 3.45, 95% CI: 2.28-5.21, p < 0.001; 37.95% morbidity rate) and mortality (OR 3.67, 95% CI: 2.15-6.27, p < 0.001) than direct presentations (OR 0.23 for morbidity, OR 0.34 for mortality), likely owing to delays in referral pathways or complex cases, consistent with Nega’s findings [25]. Older age was a significant predictor, with patients aged 58-67 years (OR 1.83 for morbidity, OR 2.97 for mortality, p < 0.05) and 78-88 years (OR 2.39 for morbidity, OR 3.79 for mortality, p < 0.05) showing increased risk; the >88 years group had a 66.67% morbidity and mortality rate, although with wide confidence intervals due to the small sample size (n = 3). Ademe et al. similarly reported higher complication and mortality rates in older patients [24]. Comorbidities (OR 12.15 for morbidity, OR 7.62 for mortality) and fever (OR 36.07 for morbidity, OR 8.27 for mortality) were strong predictors, reflecting the impact of underlying conditions (e.g., sickle cell disease) and systemic infection. Fecal fistula (OR 18.98) and sepsis (OR 10.58) were particularly lethal, especially in typhoid perforation and gangrenous bowel cases, consistent with Kotiso et al. and Nega [18,25].

Limitations

This retrospective study has several limitations. The reliance on theater records and patient case notes may introduce data incompleteness or inconsistencies, particularly for variables like clinical findings or comorbidities, due to variations in documentation practices. The assumption of predictor independence in the multivariate logistic regression analysis may overlook potential interactions, such as between sepsis and fecal fistula or fever and comorbidities, which could influence outcomes. The small sample size for patients aged >88 years results in wide confidence intervals, limiting the precision of estimates for this group. Additionally, the study did not explore specific causes of delayed presentation, such as referral system inefficiencies or patient awareness, which likely impact morbidity and mortality rates in this low-resource setting. Prospective studies with standardized data collection and adjustment for confounding interactions are needed to address these constraints.

## Conclusions

This 10-year retrospective study at Federal Teaching Hospital, Ido-Ekiti, Nigeria, highlights the significant burden of non-traumatic acute abdomen, primarily driven by acute appendicitis, intestinal obstruction, and intestinal perforation. The near-equal gender distribution and predominance of younger patients reflect regional demographic trends. Delayed presentation, comorbidities, and fever were key drivers of morbidity and mortality, worsened by complications like wound infection and sepsis. Compared to regional studies, these findings underscore the critical role of healthcare access, timely referrals, and systemic infection management in low-income settings. To improve outcomes, there is an urgent need for enhanced early diagnosis, prompt surgical intervention, and strengthened healthcare infrastructure in Nigeria and similar resource-limited environments.
